# Functional single nucleotide polymorphisms in dopaminergic receptors D2 predict clinical response to Cariprazine

**DOI:** 10.3389/fphar.2023.1182393

**Published:** 2023-05-09

**Authors:** Marco De Pieri, Marco Ferrari, Franca Marino, Rafael Traber, Emilio Bolla, Marco Cosentino

**Affiliations:** ^1^ Center for Research in Medical Pharmacology, University of Insubria, Varese, Italy; ^2^ PhD Program in Clinical and Experimental Medicine and Medical Humanities, University of Insubria, Varese, Italy; ^3^ General Psychiatry Service, Hopitaux Universitaires de Genève, Genève, Switzerland; ^4^ Cantonal Sociopsychological Organization, Ticino, Switzerland

**Keywords:** pharmacogenetics, Cariprazine, DRD2, DRD3, personalized medicine

## Abstract

Cariprazine (CAR) is an antipsychotic drug for the treatment of schizophrenia (SCZ) and bipolar disorder (BD), and it acts as a partial agonist on the dopamine receptors (DR), D2, and D3. Although many single nucleotide polymorphisms (SNPs) in genes coding for these receptors are known to influence response to antipsychotics, to date, no study on CAR pharmacogenetics exists. In this pilot study, we investigated the relationship between SNPs in DRD2 (rs1800497 and rs6277) and DRD3 (rs6280), and response to CAR treatment, evaluated by the psychometric Brief Psychiatric Rating Scale (BPRS), in a cohort of Caucasian patients. We found a significant association between DRD2 rs1800497 and rs6277 and response to CAR treatment. When genotypes were combined into an arbitrary score, the receiver operating characteristic curve analysis showed that using a cut-off value of −2.5 the response to CAR treatment could be predicted with a positive likelihood ratio of 8.0. Our study report, for the first time, a correlation between SNPs in DRD2 and response to CAR treatment. After confirmation in a larger cohort of patients, our results could open the way for the identification of new tools for the provision of response to CAR treatment.

## Introduction

Schizophrenia (SCZ) and bipolar disorder (BD) are relatively common and chronic mental disorders notable for their marked heterogeneity in the disease course, response to treatment, and variability in pharmacological interventions (Citrome et al., 2009; Leucht et al., 2009; Volavka and Citrome, 2009; Citrome et al., 2015; Citrome et al., 2016).

Cariprazine (CAR) is a antipsychotic medication approved for the treatment of adult patients with SCZ and manic or mixed episodes associated with BD, and it acts mainly as a dopamine receptor (DR) D3-and D2 partial agonist (Kiss et al., 2010; Citrome, 2013; McCormack, 2015; Stahl, 2016; Allergan, 2017; Campbell, et al., 2017). Despite the good efficacy and low side effects associated with CAR (Orzelska-Górka et al., 2022), not all patients treated with this drug achieve the therapy target and some of them show side effects (Yan et al., 2022). The observed variability could have non-genetic causes, such as the patient’s physio-pathological characteristics, multi-drug interactions, and compliance. However, it is not possible to exclude the patient’s genetic characteristics, which could influence response to CAR.

Many single nucleotide polymorphisms (SNPs) were identified in dopamine receptor genes (DR) (Magistrelli et al., 2021). Some of these are in the coding or regulatory region and are known to influence receptor expression and function (Lundstrom and Turpin, 1996; Duan, 2003; Johnson et al., 2008; Hirvonen et al., 2009; Cosentino et al., 2018) as well as the patient’s clinical conditions (Arinami, 1997; Neville et al., 2004; Doehring et al., 2009; Kang et al., 2014; Kuo et al., 2014; Ferrari et al., 2016; Comi et al., 2017).

Although there is a plausibility of the relationship between genetic variability and response to CAR, no studies have reported on this relationship. In order to investigate if SNPs in dopamine receptor genes are related to CAR response, in a cohort of Caucasian patients with an indication of CAR treatment, we evaluated the allelic frequency of SNPs in DRD2 and DRD3 in relation to treatment response.

## Methods

### Study design and patients

This is a genetic and prospective pilot study in which SCZ and BD patients were diagnosed according to the Diagnostic and Statistical Manual of Mental Disorders, fifth edition (DSM5). Patients who start therapy with CAR were consecutively recruited over a period of 24 months. All patients were recruited at the Cantonal Psychiatric Clinic of Mendrisio. The study was approved by the local ethics committee and patients were enrolled after having read and signed an informed consent form (Ethics Committee approval 2019-01366; CE3502).

In this study we include patients with SCZ and BD diagnosed according to the DSM5 diagnostic criteria (APA, 2013) and with indication to treatment with CAR in monotherapy. We excluded patients with concomitant therapy with psychotropic drugs (except benzodiazepines) and clinically significant concomitant disease states (e.g., renal failure, hepatic dysfunction, cardiovascular disease, major neurological disorders and known) or suspected non-compliance.

All patients started CAR treatment with the standard dose of 1.5 mg/day, the dosage was increased according to the guideline indications (Campbell et al., 2017). Patients were evaluated at the time of enrolment (T0) and after 8 weeks (T1) of CAR treatment.

The follow-up duration was chosen based on results of clinical trials suggesting that CAR produce onset of effect by 1–3 weeks with no difference in efficacy after 6–8 weeks of treatment (reviewed in Campbell, 2017 Haddad and Correll, 2018). Our choice is supported also by data demonstrating that CAR given for 15 days results in 92% D3 and 79% D2 receptor occupancy, an occupancy percent that is expected to balance efficacy and CAR-related tolerability (Girgis et al., 2016).

At T0, patients’ anamnestic data were collected and psychopathological conditions were evaluated by the psychometric Brief Psychiatric Rating Scale (BPRS). At T1, the psychopathological evaluation was repeated and patients were divided into two groups, responder and no-responder, according to PBRS score reductions. We have considered a cut-off for response to CAR therapy to be a reduction in BPRS scale score of at least 50% (Leucht et al., 2009) and considered patients to be in the group of no-responder if they did not achieve this result after the dose increase.

### SNPs criteria selections and genotyping

We selected a panel of SNPs in DRD2 and DRD3 to evaluate the role of genetic variants in CAR response, giving priority to those with an expected frequency in Caucasian populations of at least 10%, with evidence of functional relevance (Table 1), and/or that we showed in previous studies to be associated with clinical responses to dopaminergic agents (Ferrari et al., 2016; Comi et al., 2017).

**TABLE 1:** Dopamine receptor genes variants were included in the study. AF, allelic frequencies in Caucasian populations.

Gene	Variant	Change	MAF	Effects	Score	References
DRD2	rs1800497	2137A>G	19	Lower striatal receptor density	−1	Johnson et al. (2008)
	rs6277	957C>T	46	Decreased mRNA stability, reduced dopamine-induced receptor upregulation, and lower DRD2 expression	−1	Duan (2003)
						Hirvonen et al. (2009)
DRD3	rs6280	25G>A	34	Higher dopamine binding affinity *in vitro*, association with alcohol and heroin dependence. And Treg-induced Teff inhibition	1	Lundstrom and Turpin (1996)
						Kang et al. (2014)
						Cosentino et al. (2018)

DRD2, dopamine receptors gene D2; DRD3, dopamine receptors gene D3; MAF (%), minor allele frequencies.

Genomic DNA was extracted by Whatman FTA Elute Micro Card kit (Qiagen, Valencia, CA) as described by the manufacturer. SNPs listed were identified by pre-designed genotyping assay (ABI) using a TaqMan probe with a StepOne Real-Time PCR System (Applied Biosystems, Foster City, United States). For further detail see Supplementary Table S1.

## Statistics

Data are shown as the mean ± standard deviation (SD), unless otherwise stated. The statistical significance of the differences between groups was assessed by the Mann–Whitney U test. The χ2 test was used to assess the Hardy–Weinberg equilibrium in allele distributions. Differences in alleles frequencies were analyzed by the χ2-test for trend (or by Fisher’s exact test, as appropriate). The odds ratio (OR) with 95% confidence interval (CI) was calculated using a recessive model (wild type/heterozygous vs. homozygous).

For the analysis, genotypes of individual receptors were combined into functional categories based on published descriptions of the alleles (Table 1). To this end, an arbitrary score was defined by assigning +1 to each allele associated with increased dopaminergic firing, −1 to each allele associated with decreased dopaminergic firing, and 0 to all the other alleles. A receiver operating characteristic (ROC) curve analysis was used to assess the discrimination of patients with and without response to CAR based on the arbitrary score.

## Results

### Patients

We enrolled 20 patients diagnosed with SCZ (n = 11) or BD (n = 9) and treated with CAR (4.0 ± 1.2 mg/day). Among these, 16 patients achieved a reduction of BPRS scale score greater than 50% after 8 weeks of CAR treatment, and, therefore, were considered a responder to therapy. For four patients, the reduction of BPRS after 8 weeks of CAR treatment did not achieve the 50%, thus these patients were placed in the no-responder group. We did not find any differences in diagnosis, gender, age distribution, or drug dosage between groups, with the exception of the BPRS score that was significantly higher in the no-responder group compared to the responder group at visit T1 (Table 2).

**TABLE 2:** Characteristics of patients enrolled in the study. * = *p* < 0.001 vs. responder at T1.

	All		Responder		No-responder	
	T0	T1	T0	T1	T0	T1
Subjects numbers	20	—	16	—	4	—
Diagnosis (SCZ/BD)	11/9	—	7/9	—	4/0	—
Gender (M/F)	9/11	—	8/8	—	1/3	—
Age (mean ± DS)	36 ± 11	—	39 ± 14	—	37 ± 8.3	—
Drug dose (mg/die)	1.5 ± 0	4.0 ± 1.2	1.5 ± 0	4.0 ± 1.3	1.5 ± 0	4.1 ± 0.8
BPRS score	58.1 ± 8.8	31.7 ± 10.9	57.5 ± 9.3	27.9 ± 7.5	60.5 ± 7.0	46.0 ± 10.4*

T0, time of enrolment; T1, 8 weeks treatments; SCZ, schizophrenia; BD, bipolar disorder; M male; F, female; BPRS, brief psychiatric rating scale.

### Relationship between SNPs in DRD2, DRD3 and response to CAR

All alleles were in Hardy–Weinberg equilibrium. Table 3 shows that the allelic frequency of DRD2 rs1800497 and rs6277 was significantly higher in no-responder patients, whereas the allelic frequency of DRD3 rs6280 was not significantly different between the two groups.

**TABLE 3:** Correlations between patients’ genotype and CAR response. * = χ2-test for trend; # = Fisher’s exact test.

Gene	SNP	Genotype	Responder N (%)	No-responder N (%)	P*	P#	Odds ratio (95% C.I.)
DRD2	rs1800497	G/G	7 (43)	0 (0)	0.005	0.013	45 (2.1–938)
		G/A	8 (50)	1 (25)			
		A/A	1 (7)	3 (75)			
	rs6277	C/C	8 (50)	0 (0)	0.016	0.088	15 (0.9–251)
		C/T	7 (43)	2 (50)			
		T/T	1 (7)	2 (50)			
DRD3	rs6280	G/G	3 (19)	2 (50)	0.125	1.000	0.43 (0.02–10)
		G/A	10 (62)	2 (50)			
		A/A	3 (19)	0 (0)			

SNP, single nucleotide polymorphism; N, number of subjects, DRD2, dopamine receptors D2; DRD3, dopamine receptors D3.

### Genotype combinations

When alleles were combined by assigning an arbitrary score of +1 or −1 to each SNP associated with an increased or decreased dopamine receptor genes firing (Table 1), the combination of all SNPs resulted in a score of 0.8 ± 1.7 for the whole population. The score was −0.2 ± 1.1 for responder patients and −2.8 ± 1.0 for no-responder patients (*p* = 0.003). The median value in the whole population was −1, and 3 responder subjects (19% of all responder subjects) and all no-responder subjects (100%) had a score less than −1. The resulting OR was 34.7 (95% 1.5–810.2) (*p* = 0.007).

**FIGURE 1:**
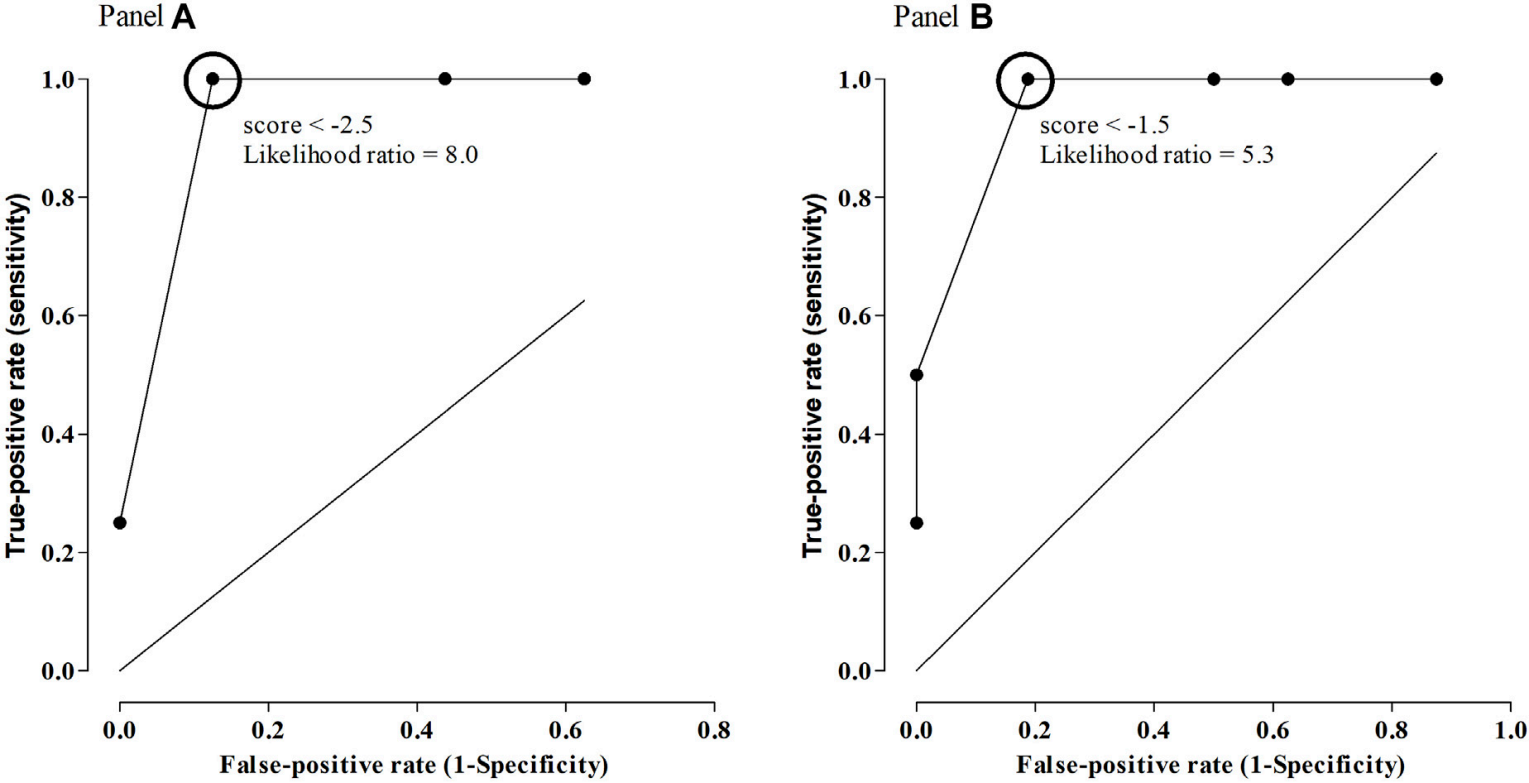
ROC curve of arbitrary scores as predictors of response to CAR, using all SNPs considered in the study [Panel **(A)**], or only SNPs in DRD2 [Panel **(B)**].

The ROC curve of the arbitrary score had an area under the curve (AUC) of 0.953 (95% 0.8615–1.045) (*p* = 0.006). Using the cut-off value of −1.5, response to CAR treatment was predicted with a specificity of 97%, a sensitivity of 81.2%, and a positive likelihood ratio of 5.33 (Figure 1A).

The same calculations were performed by combining only SNPs that were significantly associated with response to CAR (rs1800497 and rs6277). In this case, the score was −1.7 ± 1.3 for the whole population. The score was −1.2 ± 1.1 for responder patients and −3.3 ± 0.5 for no-responder patients (*p* = 0.002). The median value in the whole population was −2, and 2 responder subjects (14% of all responder subjects) and all no-responder subjects (100%) had a score less than −2. The resulting OR was 52.2 (95% 2.1–1301) (*p* = 0.003).

The ROC curve of the arbitrary score had an AUC of 0.9531 (95% 0.8634–1.043) (*p* = 0.006). Using the cut-off value of −2.5, response to CAR treatment could be predicted with a specificity of 100%, a sensitivity of 87.5%, and a positive likelihood ratio of 8.00 (Figure 1B).

## Discussion

The main result of our study is that the DRD2 rs1800497 rs6277 (but not DRD3 rs6280) are linked to response to CAR treatment in a population of Caucasian SCZ or BD patients. Moreover, the ROC curve of arbitrary score obtained by SNP-to-SNP combination showed that by using a cut-off value of −2.5 the response to CAR treatment could be predicted with high specificity and sensitivity. Although previously reported studies point out that the functional SNPs in DRD2 and DRD3 influence response to antipsychotics treatment (Suzuki et al., 2000; Ikeda et al., 2008; Kim et al., 2008; Hwang et al., 2010; Alladi et al., 2019), this is the first study, to the best of our knowledge, demonstrating such a relationship with response to CAR.

We focused on CAR pharmacodynamics, rather than its metabolism, indeed, although CAR is extensively metabolized by CYP3A4 and CYP2D6, CAR metabolites retain a significant pharmacological activity thus, in our opinion, it is unlikely that SNPs in genes coding for these enzymes could induce significant modification of CAR clinical efficacy. On the other hand, DRD2 and DRD3 play a key role in the CAR mechanism of action, and it is therefore plausible that SNPs affecting dopamine receptor genes expression/activity could, in turn, influence response to CAR.

In order to increase the chance to identify the correlation between patients’ genetic profile and response to CAR, we decided to choose only SNPs whose functional consequences were known. The, rs1800497, in the DRD2 gene is known to alter binding specificity, reduce DRD2 expression in the striatum (Johnson et al., 2008), and was associated with addiction (Arinami, 1997; Neville et al., 2004; Doehring et al., 2009). The, rs6277, is known to decrease DRD2 mRNA stability and translation, reduced dopamine-induced upregulation of DRD2 membrane expression *in vitro* (Duan, 2003), and was associated with lower receptor expression in the cortex and thalamus of healthy subjects (Hirvonen et al., 2009). Finally, the DRD3 rs6280 was associated with higher binding affinity for DRD3 selective ligands (Lundstrom and Turpin, 1996) and addiction (Kang et al., 2014; Kuo et al., 2014).

Our results showed a correlation between response to CAR treatment and SNPs in the DRD2 but not with SNPs in DRD3. This discrepancy could be explained through the different distribution and functions of dopamine receptor genes in the central nervous systems. Indeed DRD2 is the target of the mesolimbic dopaminergic pathway projecting from the ventral tegmental area to the ventral striatum, whose dysfunction is regarded as the final common pathway for the positive symptoms of psychosis, both in the context of SCZ and BD (Schwartz et al., 1995; Kapur, 2003; Stahl, 2013; Stahl, 2018). On the other hand, the role of DRD3 remains uncertain and loosely linked to cognitive functioning, emotions, and mood regulation (Stahl, 2013). For this reason, it is possible to assume that the greater relevance of SNPs in DRD2 in response to CAR could be related to the major role played by this receptor in psychiatric disease pathophysiology as well as in the activity of antipsychotic drugs.

Another interesting point emerging from our results is that when a ROC curve analysis was used to assess the response to CAR based on the arbitrary score, the results indicated that a cut-off value of −2.5 would predict response to CAR with high specificity and sensitivity, with a positive likelihood ratio of 8.00. A positive likelihood ratio greater than 5 is conventionally considered suitable for both the assessment of pre-test probability of a patient having the disease tested as well as the estimation of a post-test probability of the disease state (McGee, 2002).

In this study, patients were enrolled taking into account the indication for CAR treatment regardless of the diagnosis (SCZ or BD). However, we observed that no patients were diagnosed with BD in the no-responder patient group. This observation forces us to consider the possibility that the association between SNPs in dopamine receptor genes and response to CAR could be limited to SCZ patients. Since, the stratification of the subjects according to different diagnoses is impossible in our study due to the low number of enrolled subjects, further studies, in a greater number of patients, are required to confirm or deny the predictive role of SNPs in dopamine receptor genes for CAR response in patients with BD.

We are conscious that this study presents some limits, in particular the small number of enrolled patients. However, in this regard, it is important to note that we have selected only patients in monotherapy with CAR, so avoiding the possible confounding factor due to treatment with more drugs which is common in psychiatric disease.

In conclusion in this pilot study, we report, for the first time, a relationship between a patient’s genetic profile and response to CAR treatment. If confirmed in a larger cohort of patients, our results, although preliminary, could open the way for the identification of new, useful tools for the prevision of a response to CAR treatment in SCZ and BD patients, and ultimately allow the possibility for a target therapy in patients with indication to treatment with this drug.

## Data Availability

The original contributions presented in the study are included in the article/Supplementary Material further inquiries can be directed to the corresponding author.
