# Supplementary material for: Functional single nucleotide polymorphisms in dopaminergic receptors D2 predict clinical response to Cariprazine
Source: Front Pharmacol. 2023 May 9;14:1182393. doi: 10.3389/fphar.2023.1182393 (PMC10203397; doi:10.3389/fphar.2023.1182393)
Supplement: Supplementary file 1 [file Table4.docx]

| **Table S1.** TaqMan pre-designed primers/probe details (**Panel A**) and Thermal Cycling Conditions (**Panel B**). | | | | | |
| --- | --- | --- | --- | --- | --- |
| **Panel A** | | | | | |
| **Gene** | **SNP** | **Context Sequence [VIC/FAM]** | | | |
| *DRD2* | rs1800497 | CACAGCCATCCTCAAAGTGCTGGTC**[A/G]**AGGCAGGCGCCCAGCTGGACGTCCA | | | |
|  | rs6277 | TCTTCTCTGGTTTGGCGGGGCTGTC**[G/A]**GGAGTGCTGTGGAGACCATGGTGGG | | | |
|  |  |  | | | |
| *DRD2* | rs6280 | GCCCCACAGGTGTAGTTCAGGTGGC**[C/T]**ACTCAGCTGGCTCAGAGATGCCATA | | | |
|  |  |  | | | |
| **Panel B** | | | | | |
| **Stage** | | | **Temperature** | **Stage Duration** | **Number of cycles** |
| Taq activation | | | 95°C | 10 minutes | 1 |
| Amplification (Annealing/Extension) | | | 95° C  60° C | 15 seconds  60 seconds | 40 |
| Read Stage | | | 60 °C | 30 seconds | 1 |
